# Supplementary material for: Evaluating Different Extraction Approaches for GC-MS Based Metabolomics Analysis of the Giant Pandas’ Fur
Source: Toxics. 2022 Nov 14;10(11):688. doi: 10.3390/toxics10110688 (PMC9696619; doi:10.3390/toxics10110688)
Supplement: Supplementary file 1 [file toxics-10-00688-s001.zip › toxics-1993026-supplementary.pdf]

toxics MDPI

Supplementary Materials: Evaluating Different Extraction Approaches for GC-MS Based Metabolomics Analysis of the Giant Pandas' Fur

Yang Yang [1,2,3,4,†], Yanqiang Yin [5,†], Xianglan Tang [6], Yinyin Xia [7], Jinya Zhang [8], Chun Yan [8], Weixuan Zhang [8], Hua Zhang [1,2,3,4,*] and Ting-Li Han [2,3,4,6,*]

1 Department of Obstetrics and Gynecology, The First Affiliated Hospital of Chongqing Medical University, Chongqing 400016, China
2 Ministry of Education of China International Collaborative Joint Laboratory of Reproduction and Development, Chongqing Medical University, Chongqing 400000, China
3 State Key Laboratory of Maternal and Fetal Medicine of Chongqing Municipality, Chongqing Medical University, Chongqing 400000, China
4 Institute of Life Sciences, Chongqing Medical University, Chongqing 400000, China
5 Chongqing Zoo, Chongqing 400050, China
6 Department of Obstetrics and Gynecology, The Second Affiliated Hospital of Chongqing Medical University, Chongqing 400010, China
7 School of Public Health and Management, Chongqing Medical University, Chongqing 400000, China
8 School of Clinical Medicine, Chongqing Medical University, Chongqing 400000, China
* Correspondence: zh2844@gmail.com (H.Z.); tinglinghan@cqmu.edu.cn (T.-L.H.)
† These authors contributed equally to this work.

Table S1. Number of identified metabolites and CVs of each biochemical compound class identified in giant panda furs comparing across eight different extraction methods.

Biochemical Classes	HCL+ACN		HCL+Hexane-EtOAc 1		HCL+Hexane-EtOAc 4		HCL+MeOH		NaOH+ACN		NaOH+Hexane-EtOAc 1		NaOH+Hexane-EtOAc 4		NaOH+MeOH	
	#	CV±SD	#	CV±SD	#	CV±SD	#	CV±SD	#	CV±SD	#	CV±SD	#	CV±SD	#	CV±SD
Alkanes	1	12.4	1	7.9	1	12.2	1	3.4	1	14.7	1	7.6	1	40.7	1	9.9
Amino acids	18	5.2±7.5	18	4.5±4.4	18	4.2±4.8	18	3.4±3.2	16	4.8±2.3	17	7.2±5.3	16	24.1±14.0	16	5.5±4.9
Amino acids and derivatives	10	6.4±4.6	9	6.1±3.4	9	4.7±3.1	7	4.8±3.2	9	10.0±3.6	8	14.0±8.6	8	22.0±13.0	7	9.4±4.4
Organic acids	14	9.9±5.1	15	7.8±4.0	11	10.8±5.3	12	11.0±5.0	15	12.1±6.8	8	9.2±2.8	10	32.0±13.7	15	9.2±5.6
Saturated fatty acids	2	7.4±1.8	1	5.0	1	1.8	2	7.7±1.5	6	7.9±4.8	5	6.1±2.8	6	41.9±2.1	6	6.7±3.2
Secondary metabolites	3	7.4±1.9	3	9.0±1.9	3	8.6±6.8	2	16.5±2.2	2	8.3±1.7	2	9.3±3.4	2	62.3±2.1	2	13.2±6.4
Unsaturated fatty acids	2	9.2±5.9	2	12.9±3.1	1	10.4	1	13.4	2	4.7±3.4	2	12.2±1.8	2	29.1±12.5	2	5.7±1.9
TCA cycle and derivatives	3	7.1±4.5	3	7.8±3.9	3	14.1±5.1	3	11.8±8.2	3	13.4±3.3	3	10.2±1.9	3	28.6±16.3	3	9.2±6.0

Table S2. The semi-quantitative log values of identified metabolites in giant panda furs across the eight different extraction methods.

Name	Classfication6b	HCL+ACN	HCL+Hexane-EtOAc 1	HCL+Hexane-EtOAc 4	HCL+MEOH	NAOH+ACN	NAOH+Hexane-EtOAc 1	NAOH+Hexane-EtOAc4	NAOH+MEOH
10,13-dimethyltetradecanoic acid (C17_0)	Saturated fatty acids	3.95±0.06	3.84±0.02	3.88±0.01	3.94±0.02	4.62±0.02	4.65±0.02	4.72±0.20	4.85±0.03
1-Aminocyclopropane-1-carboxylic acid	Organic acids	4.58±0.11	4.44±0.03	4.51±0.11	4.51±0.08	3.64±0.02	3.48±0.05	NA	3.71±0.02
2,4-Di-tert-butylphenol	Organic acids	3.70±0.05	3.61±0.05	3.65±0.03	3.71±0.05	3.69±0.09	NA	3.77±0.20	3.73±0.04
2-Aminoadipic acid	Organic acids	3.96±0.04	3.86±0.01	3.94±0.03	3.94±0.03	NA	NA	NA	NA
2-Aminobutyric acid	Organic acids	3.59±0.02	3.54±0.04	3.54±0.02	NA	4.45±0.02	4.19±0.03	4.01±0.05	4.38±0.02
2-Aminophenylacetic acid	Organic acids	NA	NA	NA	NA	3.53±0.08	NA	NA	3.57±0.10
2-Hydroxyisobutyric acid	Organic acids	NA	NA	NA	NA	3.21±0.07	NA	NA	3.06±0.06
2-ketoglutamarate	Amino acids and derivatives	6.35±0.02	NA	6.32±0.01	6.35±0.02	NA	NA	NA	NA
2-Oxoadipic acid	TCA cycle and derivatives	3.42±0.04	NA	NA	NA	NA	NA	NA	NA
2-Oxoglutaric acid	TCA cycle and derivatives	4.85±0.01	5.76±0.01	3.82±0.06	4.49±0.06	3.35±0.07	NA	NA	3.34±0.06
3-Methyl-2-oxopentanoic acid	Organic acids	3.47±0.06	3.54±0.06	3.57±0.05	NA	NA	NA	NA	NA
4-Aminobutyric acid (GABA)	Organic acids	NA	NA	NA	NA	3.71±0.05	3.68±0.03	3.73±0.15	3.73±0.03
4-Hydroxyphenylacetic acid	Organic acids	NA	NA	NA	NA	3.65±0.02	3.45±0.04	3.48±0.15	3.60±0.02
4-Hydroxyphenylethanol	Organic acids	NA	6.10±0.01	NA	NA	NA	NA	NA	NA
4-Methyl-2-oxopentanoic acid	Organic acids	4.26±0.05	4.29±0.03	4.28±0.06	4.30±0.04	NA	NA	NA	NA
Alanine	Amino acids	6.52±0.01	6.45±0.01	6.47±0.01	6.47±0.01	5.85±0.01	5.58±0.01	5.32±0.01	5.81±0.01
Asparagine	Amino acids	4.19±0.15	4.11±0.06	4.14±0.09	4.28±0.05	NA	NA	3.43±0.17	NA
Aspartic acid	Amino acids	6.54±0.01	6.49±0.01	6.51±0.01	6.54±0.01	5.57±0.03	5.40±0.01	5.03±0.16	5.53±0.02
Benzoic acid	Organic acids	3.20±0.04	NA	NA	3.14±0.07	3.11±0.03	NA	NA	3.06±0.03
Carbamic acid	Organic acids	3.64±0.06	NA	NA	NA	NA	NA	NA	NA
Caprylic acid	Organic acids	NA	NA	NA	NA	NA	NA	2.93±0.18	NA
cis-4-Hydroxyproline	Amino acids and derivatives	4.79±0.02	4.77±0.04	NA	4.81±0.02	NA	NA	NA	NA
Citraconic acid	Unsaturated fatty acids	4.10±0.16	4.11±0.06	NA	NA	3.92±0.03	3.84±0.05	3.73±0.09	3.95±0.03
Citramalic acid	Organic acids	NA	NA	NA	NA	3.85±0.02	3.80±0.03	3.77±0.14	3.89±0.02
Citric acid	TCA cycle and derivatives	NA	3.75±0.04	3.80±0.03	NA	NA	4.47±0.05	4.60±0.17	NA
Creatinine	Secondary metabolites	4.86±0.06	4.75±0.05	4.84±0.06	4.82±0.06	NA	NA	3.34±0.28	NA
Cysteine	Amino acids	5.52±0.03	5.37±0.07	5.45±0.03	5.52±0.03	5.23±0.01	4.75±0.04	4.32±0.22	5.23±0.02
D_Citraconic acid	Unsaturated fatty acids	4.80±0.08	4.83±0.04	5.10±0.04	5.23±0.05	NA	NA	NA	NA
D_Fumaric acid	TCA cycle and derivatives	NA	NA	NA	3.35±0.08	3.69±0.04	3.69±0.04	3.52±0.04	3.69±0.02
Dimethyl aminomalonic acid	Organic acids	4.82±0.09	4.73±0.02	4.82±0.05	4.77±0.05	4.43±0.03	4.50±0.03	4.11±0.19	3.27±0.04
DL-gamma-methyl-ketoglutaramate	Organic acids	4.11±0.03	3.93±0.03	3.92±0.05	4.05±0.01	3.49±0.07	NA	3.57±0.12	3.56±0.07
Fumaric acid	TCA cycle and derivatives	NA	NA	NA	NA	NA	NA	NA	NA
Glutamic acid	Amino acids	6.32±0.02	6.27±0.02	6.28±0.01	6.31±0.02	5.42±0.03	5.15±0.01	4.83±0.12	5.37±0.04
Glutaric acid	Organic acids	NA	NA	NA	NA	3.35±0.04	NA	NA	3.29±0.02
Glutathione	secondary metabolites	5.60±0.04	5.51±0.03	5.57±0.01	5.56±0.07	4.76±0.03	4.41±0.04	NA	4.71±0.07
Glycine	Amino acids	6.52±0.01	6.45±0.01	6.48±0.01	6.49±0.01	6.18±0.02	6.04±0.01	5.77±0.04	6.17±0.01
Heneicosanoic acid (C21_0)	Saturated fatty acids	NA	NA	NA	NA	4.05±0.02	4.14±0.01	4.18±0.17	4.37±0.02
Histidine	Amino acids	5.08±0.01	5.03±0.02	5.04±0.02	5.06±0.02	4.03±0.04	3.46±0.03	NA	3.90±0.01

Homocysteine	Amino acids and derivatives	4.63±0.03	4.63±0.02	4.62±0.02	4.62±0.01	NA	NA	NA	NA
Hydroxybenzoic acid	Organic acids	3.63±0.03	3.49±0.01	3.48±0.01	3.60±0.05	3.25±0.03	NA	NA	3.27±0.03
Isoleucine	Amino acids	6.67±0.01	6.63±0.01	6.64±0.01	6.65±0.01	4.69±0.01	4.39±0.06	3.99±0.05	4.63±0.03
Lactic acid	secondary metabolites	3.96±0.05	3.87±0.03	3.89±0.03	NA	5.09±0.04	4.64±0.03	4.03±0.29	4.96±0.03
Leucine	Amino acids	6.67±0.01	6.63±0.01	6.64±0.01	6.65±0.01	4.84±0.01	4.67±0.02	4.40±0.04	4.80±0.01
Levulinic acid	Organic acids	4.30±0.01	4.19±0.02	4.21±0.05	4.08±0.01	NA	NA	NA	NA
Lysine	Amino acids	6.35±0.02	6.29±0.02	6.32±0.01	6.35±0.02	4.84±0.01	4.41±0.03	3.98±0.12	4.81±0.01
Malic acid	TCA cycle and derivatives	NA	NA	NA	NA	NA	NA	NA	NA
Malonic acid	Organic acids	NA	4.03±0.05	NA	4.10±0.05	NA	NA	NA	NA
Margaric acid (C17_0)	Saturated fatty acids	NA	NA	NA	NA	3.67±0.06	3.71±0.02	3.75±0.19	3.92±0.02
Methionine	Amino acids	5.59±0.01	5.55±0.01	5.57±0.01	5.57±0.01	NA	4.26±0.01	NA	NA
Myristic acid (C14_0)	Saturated fatty acids	NA	NA	NA	NA	3.49±0.01	3.45±0.04	3.51±0.18	3.59±0.01
N-(Carboxymethyl)-L-alanine	Amino acids and derivatives	4.74±0.07	4.66±0.04	4.65±0.02	NA	NA	NA	NA	3.30±0.04
N-Acetylcysteine	Amino acids and derivatives	NA	NA	NA	NA	3.40±0.03	3.86±0.09	3.76±0.03	NA
N-Acetylglutamic acid	Amino acids and derivatives	NA	NA	NA	NA	3.89±0.02	4.45±0.07	4.34±0.03	NA
NADP_NADPH	Organic acids	4.56±0.04	4.36±0.02	4.43±0.02	4.48±0.02	3.48±0.05	3.66±0.02	3.48±0.13	3.41±0.01
Nicotinamide	Organic acids	NA	3.12±0.03	NA	3.23±0.06	NA	NA	NA	NA
Norleucine	Amino acids and derivatives	4.91±0.02	4.81±0.01	4.82±0.01	4.89±0.01	4.13±0.04	4.02±0.04	3.87±0.05	4.16±0.05
Norvaline	Amino acids and derivatives	5.12±0.01	5.79±0.01	5.03±0.01	5.12±0.02	4.63±0.06	NA	4.10±0.17	4.66±0.06
O-Acetylserine	Amino acids and derivatives	NA	NA	NA	NA	3.35±0.08	4.10±0.13	4.33±0.07	NA
Ornithine	Amino acids and derivatives	4.34±0.03	4.30±0.02	4.32±0.02	4.39±0.04	5.17±0.03	4.85±0.03	4.51±0.11	5.13±0.02
Oxaloacetic acid	TCA cycle and derivatives	4.10±0.02	NA	NA	NA	NA	NA	NA	NA
Pentadecanoic acid (C15_0)	Saturated fatty acids	NA	NA	NA	NA	3.28±0.05	NA	NA	3.41±0.02
Phenylalanine	Amino acids	6.29±0.01	6.24±0.01	6.26±0.01	6.28±0.01	5.04±0.01	4.93±0.01	4.47±0.12	4.99±0.01
Proline	Amino acids	6.92±0.01	6.91±0.01	6.92±0.01	6.92±0.01	6.22±0.01	5.94±0.01	5.60±0.03	6.16±0.01
Pyroglutamic acid	Amino acids and derivatives	6.24±0.01	6.17±0.02	6.20±0.01	6.23±0.01	5.33±0.04	5.11±0.02	NA	5.32±0.02
Pyruvic acid	Organic acids	NA	NA	NA	NA	3.01±0.12	NA	3.10±0.24	NA
Serine	Amino acids	5.79±0.01	5.67±0.01	5.72±0.01	5.76±0.01	5.09±0.02	4.75±0.09	4.60±0.06	5.00±0.08
Stearic acid (C18_0)	Saturated fatty acids	3.52±0.03	NA	NA	3.58±0.03	3.95±0.03	4.04±0.02	4.09±0.19	4.24±0.05
Succinic acid	TCA cycle and derivatives	4.10±0.05	4.07±0.04	4.10±0.08	4.01±0.01	3.99±0.06	3.88±0.03	4.01±0.17	3.97±0.02
tert-Leucine	Amino acids and derivatives	5.82±0.01	5.76±0.01	5.78±0.01	NA	5.16±0.03	4.66±0.06	4.28±0.13	4.92±0.02
Threonine	Amino acids	6.17±0.01	6.12±0.01	6.15±0.01	6.16±0.01	4.90±0.01	4.66±0.02	4.47±0.13	4.91±0.01
trans-4-Hydroxyproline	Amino acids and derivatives	3.54±0.02	3.38±0.04	3.47±0.05	NA	3.92±0.05	3.66±0.05	3.71±0.19	3.90±0.07
trans-Cinnamic acid	Organic acids	NA	3.32±0.02	NA	NA	NA	3.65±0.06	3.43±0.03	3.34±0.03
trans-Vaccenic acid	Unsaturated fatty acids	NA	NA	NA	NA	3.88±0.01	3.84±0.04	3.85±0.17	4.00±0.01
Tryptophan	Amino acids	5.13±0.02	5.07±0.01	5.08±0.02	5.28±0.01	4.66±0.01	4.52±0.03	3.98±0.14	4.56±0.01
Tyrosine	Amino acids	6.16±0.01	6.10±0.01	6.13±0.01	6.17±0.01	5.48±0.02	5.03±0.02	3.49±0.21	5.43±0.01
Valine	Amino acids	6.55±0.01	6.48±0.01	6.51±0.01	6.52±0.01	4.80±0.03	4.56±0.03	4.32±0.05	4.78±0.03
